# Cervical Alignment of Patients with Basilar Invagination: A Radiological Study

**DOI:** 10.1111/os.13212

**Published:** 2022-02-13

**Authors:** Jun‐yu Lin, Ming‐gui Bao, Shao‐yi Lin, Jun‐hao Liu, Qi Liu, Ruo‐yao Li, Zu‐cheng Huang, Qing‐an Zhu, Zhong‐min Zhang, Wei Ji

**Affiliations:** ^1^ Division of Spinal Surgery, Department of Orthopaedics Nanfang Hospital, Southern Medical University Guangzhou China; ^2^ Department of Orthopaedics and Traumatology, LKS Faculty of Medicine The University of Hong Kong Hong Kong City Hong Kong

**Keywords:** Basilar invagination, C_0_‐C_2_ angle, Cervical alignment, Cervical curvature, Dynamic X‐ray images

## Abstract

**Objective:**

To investigate the cervical alignment and the relative range of motion (ROM) in patients with basilar invagination (BI).

**Methods:**

A total of 40 BI cases (38.1 years old ± 17.9 years old, 19 male and 21 female) and 80 asymptomatic individuals (33.8 years old ± 10.8 years old, 40 male and 40 female) were included. The Skull‐C_2_/Skull‐BV, Skull‐C_7_, C_2_‐C_7_/BV‐C_7_ wall angles, C_0_‐C_2_/C_0_‐BV, C_0_‐C_7_, C_1_‐C_7_, and C_2_‐C_7_/BV‐C_7_ angles were measured in dynamic X‐ray images (including neutral, extension, and flexion positions). Correlation between the upper and lower cervical curvatures were analyzed. The total, extension, and flexion ROMs of these angles were calculated, respectively.

**Results:**

The BI patients had a smaller C_0_‐C_2_/C_0_‐BV angle (18.2° ± 16.4° *vs* 30.9° ± 9.3°), but larger C_2_‐C_7_/BV‐C_7_ (32.2° ± 16.1° *vs* 19.4° ± 10.6°) and C_2_‐C_7_/BV‐C_7_ wall angles (37.8° ± 17.2° *vs* 23.6° ± 10.2°) than the control group in neutral position. The upper and lower curvatures correlated negatively in neutral (*r* = −0.371), extension (*r* = −0.429), and flexion (*r* = −0.648) positions among BI patients, as well as in extension position (*r* = −0.317) among control group. The BI patients presented smaller total ROMs in Skull‐C_2_/Skull‐BV (12.3° ± 16.6° *vs* 19.7° ± 10.9°), C_0_‐C_2_/C_0_‐BV (8.1° ± 11.1° *vs* 17.6° ± 10.5°), and C_0_‐C_7_ angles (57.8° ± 14.2° *vs* 78.3° ± 17.9°), but a larger total ROM in C_2_‐C_7_/BV‐C_7_ wall angle (52.8° ± 13.9° *vs* 27.0° ± 16.1°) than the control group. The BI patients also presented smaller extension ROMs in Skull‐C_2_/Skull‐BV (6.9° ± 9.4° *vs* 12.5° ± 9.3°), Skull‐C_7_ (24.5° ± 10.9° *vs* 30.7° ± 12.5°), and C_0_‐C_2_/C_0_‐BV angles (4.4° ± 7.8° *vs* 9.9° ± 8.6°) than the control group. Moreover, the BI patients showed smaller absolute values of flexion ROMs in Skull‐C_2_/Skull‐BV (−5.2° ± 9.4° *vs* −7.3° ± 8.0°), C_0_‐C_2_/C_0_‐BV (−3.2° ± 8.8° *vs* −7.7° ± 8.7°), and C_0_‐C_7_ angles (−33.2° ± 13.0° *vs* −52.8° ± 19.2°), but a larger absolute value of flexion ROM in C_2_‐C_7_/BV‐C_7_ wall angle (−33.9° ± 14.8° *vs* −8.2° ± 15.1°).

**Conclusion:**

The cervical spine was stiffer in BI patients than the asymptomatic individuals, especially in the upper cervical curvature. The negative correlation between upper and lower cervical curvatures was more obvious in BI patients.

## Graphical abstract

This study investigated the cervical alignment of patients with basilar invagination (BI), finding the cervical spine was stiffer in BI patients than the asymptomatic individuals, especially in the upper cervical curvature. Moreover, the upper and lower cervical curvatures correlated negatively in neutral, extension, and flexion positions among BI patients.

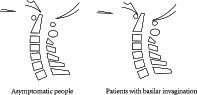

## Introduction

The basilar invagination (BI) is a congenital or degenerative deformity of the craniocervical junction (CVJ) region, with the odontoid prolapsed into the foramen magnum, leading to compression of structures in the skull base^1^, ^2^. Patients are diagnosed with BI when the odontoid process surpasses above the Chamberlain line (from the hard palate to the opisthion) for more than 5 mm^3^.

BI patients are commonly accompanied with Chiari malformation, syringomyelia or atlantoaxial dislocation in clinical practice. Accordingly, Goel *et al*. categorized the BI patients into Group I as patients without Chiari malformation and Group II as patients with presence of Chiari malformation^4^. The odontoid process of dentata was dislocated from the anterior arch of atlas in Group I, while the clivus, odontoid process, and anterior arch remained well‐aligned in Group II. Further, a new classification was developed by Goel in 2004. In brief, those with the odontoid process above the Chamberlain, McRae, and Wackenheim lines were categorized as Group A, while those with the odontoid process above the Chamberlain line and below the McRae and Wackenheim lines were categorized as Group B^5^. Although the characteristics of different types of BI patients had been described, the features of cervical curvatures of this population were not included in the above classifications.

The cervical sagittal alignment and range of motion (ROM) can be used to depict the motor function of the cervical spine. The cervical spine has the widest ROM, and the complex structure makes it vulnerable to series of disorders and complications; thus, the sagittal alignment should be taken into consideration in surgical planning^6^. Hence, the cervical sagittal alignment has been investigated and applied in various diseases. Indicators including the C_2_‐C_7_ lordosis and occipito‐cervical angle were used to predict or assess the clinical outcomes in adjacent segment degeneration (ASD)^7^ and cervical ossification of the posterior longitudinal ligament (OPLL)^8^. Other indicators like C_2_‐C_7_ sagittal vertical axis (C_2_‐C_7_ SVA) were reported as predictors of cervical spondylotic myelopathy^9^. The correlation between craniocervical alignment (O‐C_2_ angle and C_2_‐C_7_ angle) and the development of dysphagia after surgical treatment in BI patients was also discussed, finding that the change in the O‐C_2_ angle was significantly lower in patients with postoperative dysphagia than in patients without^10^. Another study revealed that the stiffer O‐C_1_ angle, which was measured and calculated in flexion and neutral positions, indicated the dysfunction of atlanto‐occipital joint and correlated with risk of cervical spondylosis^11^. However, to the best of our knowledge, the cervical curvature and the sagittal alignment changes in neutral, extension, and flexion positions in BI patients have not been reported.

In our previous studies, a clivus plate fixation system was proposed and proved to be effective in stabilizing the defected CVJ region *via* biomechanical experiments^12^, ^13^. In order to estimate the application feasibility of the system, our team have described the characteristics of clivus and upper cervical vertebrae in asymptomatic patients via computerized tomography (CT)^14^, as well as the anatomic parameters of basilar plexus and vertebrobasilar artery adjacent to the CVJ region *via* computerized tomography angiography (CTA) and magnetic resonance imaging (MRI)^15^. We further investigated the morphology of cranial‐cervical spinal canal in BI patients, finding that the BI patients presented a shorter anteroposterior diameter, but a wider transversal diameter at the upper cranial‐cervical spinal canal^16^. In addition, our previous research revealed that the BI patients had a thinner occipital bone than control group, which might increase the risk of screw penetration in surgical fixation^17^.

Therefore, the present study aims to: (i) investigate the cervical sagittal alignment in BI patients and compare the series of Cobb angles with those in control group in neutral, extension, and flexion positions; (ii) calculate the corresponding total, extension, and flexion ROMs of various cervical angles in BI patients; (iii) investigate the correlation between the upper cervical curvatures and the lower ones in both BI patients and asymptomatic population.

## Methods

### BI Patients and Asymptomatic Population

This is a retrospective study approved by the Ethics Committee of Nanfang Hospital. A total of 40 BI patients and 80 asymptomatic people from January 2010 to December 2020 in our hospital were included. The main inclusive criteria were: (i) patients diagnosed as BI; (ii) asymptomatic population without congenital malformation at craniocervical junction region; (iii) subjects with completed preoperative cervical dynamic X‐ray images (including neutral, extension, and flexion positions); (iv) subjects without surgical history on clivus, occipital, and cervical spine; (v) the anatomic structures could be clearly identified in the radiological images. The main exclusive criteria were: (i) subjects with inflammation, tumors, or implants on the craniocervical junction region; (ii) subjects with dynamic X‐ray images where the anatomic structures were too vague to be identified or partly missed on the images. A total of 19 males and 21 females with BI met the criterion, while 40 males and 40 females from the asymptomatic population were included as a control group.

### Measurement of Cobb Angles

The Toshiba X‐ray radiograph machine was used to take the dynamic X‐ray images. All the subjects were required to stand up with their shoulders relaxed and hands dropped naturally. In neutral position, their eyes should horizontally look straight forward. In the extension/flexion position, they were requested to look up/down as best as they could. All images were exported and analyzed by RadiAnt Viewer software (Version 5.5.1).

**Fig. 1:**
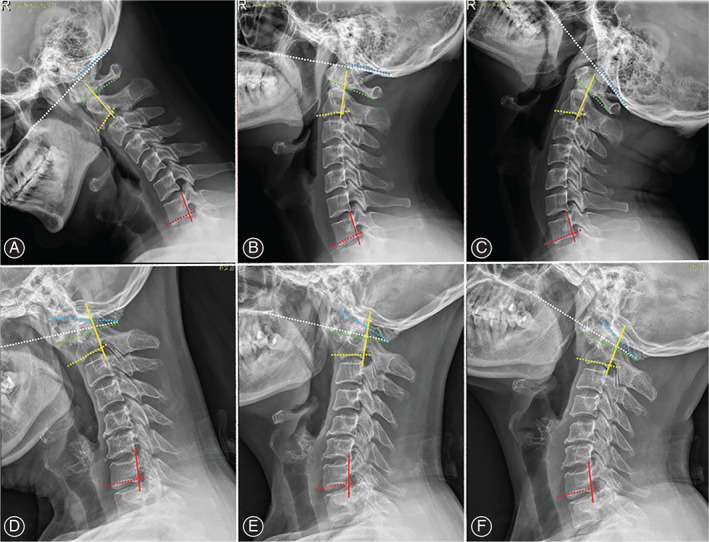
Representative dynamic X‐ray images in asymptomatic people (upper row) and BI patients (lower row) in flexion (A and D), neutral (B and E), and extension (C and F) positions. There is a fusion of posterior arch of atlas and the occipital in this BI patient, so that the Chamberlain line, C_0_ line, and C_1_ line ended at the same point. White dotted line: Chamberlain line, from the hard palate to posterior margin of foramen magnum; Blue dotted line: C_0_ line, from the anterior to posterior margin of foramen magnum; Green dotted line: C_1_ line, from the inferior margin of anterior arch to inferior margin of posterior arch of atlas; Yellow dotted line: C_2_ line, paralleled with the inferior endplate of C_2_; Red dotted line: C_7_ line, paralleled with the inferior endplate of C_7_; Yellow solid line: C_2_ wall line, paralleled with the back wall of C_2_ vertebra; Red solid line: C_7_ wall line, paralleled with the back wall of C_7_ vertebra

As shown in Figure 1, the anatomic structures were identified on the cervical dynamic X‐ray images and corresponding lines were defined: (i) Chamberlain line: from the hard palate to posterior margin of foramen magnum; (ii) C_0_ line: from the anterior to posterior margin of foramen magnum; (iii) C_1_ line: from the inferior margin of anterior arch to inferior margin of posterior arch of atlas; (iv) C_2_ line: paralleled with the inferior endplate of C_2_; (v) C_7_ line: paralleled with the inferior endplate of C_7_; (vi) C_2_ wall line: paralleled with the back wall of C_2_ vertebral body; (vii) C_7_ wall line: paralleled with the back wall of C_7_ vertebral body; in addition, the block vertebrae (BV), which is a congenital fusion of vertebral bodies with/without fusion of posterior structures, is commonly observed in BI patients according to our experience. If a BV occurred, then we defined (viii) BV line: paralleled with the inferior endplate of BV; (ix) BV wall line: paralleled with the back wall of BV vertebral body.

The Cobb angles of sagittal cervical alignment were further defined and summarized in Table 1. If there was not any BV in the BI patients, the Skull‐C_2_, Skull‐C_7_, C_2_‐C_7_ wall angles, C_0_‐C_2_, C_0_‐C_7_, C_1_‐C_7_, C_2_‐C_7_ angles would be measured on neutral, extension, and flexion X‐ray images. If a BV occurred, the Skull‐BV, Skull‐C_7_, BV‐C_7_ wall angles, C_0_‐BV, C_0_‐C_7_, C_1_‐C_7_, BV‐C_7_ angles would be measured.

**TABLE 1:** Definition of Cobb angles on dynamic X‐ray images

Angles	Definitions
Skull‐C_2_ angle	Cobb angle between Chamberlain line and C_2_ line
Skull‐C_7_ angle	Cobb angle between Chamberlain line and C_7_ line
C_2_‐C_7_ wall angle	Cobb angle between C_2_ wall line and C_7_ wall line
C_0_‐C_2_ angle	Cobb angle between C_0_ line and C_2_ line
C_0_‐C_7_ angle	Cobb angle between C_0_ line and C_7_ line
C_1_‐C_7_ angle	Cobb angle between C_1_ line and C_7_ line
C_2_‐C_7_ angle	Cobb angle between C_2_ line and C_7_ line
Skull‐BV angle	Cobb angle between Chamberlain line and BV line
BV‐C_7_ wall angle	Cobb angle between BV wall line and C_7_ wall line
C_0_‐BV angle	Cobb angle between C_0_ line and BV line
BV‐C_7_ angel	Cobb angle between BV line and C_7_ line

The CT images and reversed X‐ray images of cervical spine were used to help to determine the anatomic structures. The cases whose structures were difficult to be identified would be excluded. In situations where the fusion occurred between the atlas and occipital: (a) if both anterior and posterior arches of atlas were fused, they would be measured as anterior and posterior margin of foramen magnum, respectively, and there would be no more C_0_‐C_1_ angle and C_1_‐C_7_ angle; (b) if the anterior (or posterior) arch of atlas was fused, it would be measured as anterior (or posterior) margin of foramen magnum, but the C_0_‐C_1_ angle and C_1_‐C_7_ angle would still be measured. For all the angles, positive values indicated lordotic cervical posture (mostly in neutral and extension positions), while negative values indicated kyphotic cervical posture (mostly in flexion position).

### The Total, Extension, and Flexion ROMs

The Cobb angles were utilized to calculate the ROMs of cervical spine. The total ROM was calculated as the angles in extension position minus the angles in flexion position. The extension ROM was calculated as the angles in extension position minus the angles in neutral position. The flexion ROM was calculated as the angles in flexion position minus the angles in neutral position. Differences in the total, extension, and flexion ROMs between BI patients and asymptomatic people were analyzed, respectively.

### Correlation Between Angles

The correlation between each other among the Skull‐C_2_/Skull‐BV, Skull‐C_7_, C_2_‐C_7_/BV‐C_7_ wall angles, C_0_‐C_2_/C_0_‐BV, C_0_‐C_7_, C_1_‐C_7_, and C_2_‐C_7_/BV‐C_7_ angles were analyzed in neutral, extension, and flexion positions, respectively.

### Statistics

The software SPSS (v19.0, Chicago, IL) was used to perform statistical analyses. The Normality test (Shapiro–Wilk and Kolmogorov–Smirnova test) was firstly conducted for all parameters. The Student's *t* test and Mann–Whitney *U* test were further utilized in different variables. The repeatability test was used to compare the differences of the indicators measured in neutral, extension, and flexion positions, and the Bonferroni's multiple comparisons test was further used in multiple comparison. The Pearson correlation test was used to analyze the relationship between Cobb angles from the same position. All results were presented as mean ± standard deviation (SD). One decimal place was reserved for all the Cobb angles and ROMs. Statistical difference was set with the *α* level at 0.05.

## Results

A total of 80 asymptomatic people (half male and female, 33.8 years old ± 10.8 years old) and 40 BI patients (19 male and 21 female, 38.1 years old ± 17.9 years old) were included in the final analyses. There were 33 out of 40 cases that presented atlantoaxial dislocation in the BI group. All the BI patients complained about neck pain and 35 out of 40 BI patients had the symptom of limb weakness. The BV occurred at C_2‐3_ level in 19 cases, at C_3‐4_ level in one case, at C_2‐3‐4_ level in one case, and 19 cases had no BV in the BI group. Ten cases presented fusion in both anterior arch and clivus and posterior arches and occipital, 13 cases presented fusion only in posterior arch and occipital, one case presented fusion only in anterior arch and clivus, and seven cases presented no fusion between C_0_ and C_1_ in the BI group. No dislocation, BV, or C_0‐1_ fusion was observed in the control group. Some indicators were unavailable in few individuals since the anatomic structures were not included in the X‐ray images.

### Cervical Alignment

The results of cervical curvature of BI patients and the asymptomatic people on dynamic cervical X‐ray images were summarized in Table 2. In the neutral position, the BI patients presented smaller Skull‐C_2_/Skull‐BV angle (4.9° ± 14.6° *vs* 17.1° ± 7.9°, *P* = 0.000) and C_0_‐C_2_/C_0_‐BV angle (18.2° ± 16.4° *vs* 30.9° ± 9.3°, *P* = 0.000), but lager C_2_‐C_7_/BV‐C_7_ wall angle (37.8° ± 17.2° *vs* 23.6° ± 10.2°, *P* = 0.000) and C_2_‐C_7_/BV‐C_7_ angle (32.2° ± 16.1° *vs* 19.4° ± 10.6°, *P* = 0.000) than the control group.

**TABLE 2:** Summary of cervical alignment in BI patients and control population (unit: °)

Positions	Angles	BI group					Control group					*t*	*z*	*p*‐value*
		*N*	Min	Max	Mean	SD	*N*	Min	Max	Mean	SD			
Neutral	Skull‐C_2_/Skull‐BV	39	−35.1	43.8	4.9	14.6	80	1.2	43.6	17.1	7.9	/	−5.446	0.000
	Skull‐C_7_	39	−22.4	63.7	30.6	17.4	80	15.0	75.7	35.4	10.7	/	−1.339	0.181
	C_2_‐C_7_/BV‐C_7_ wall	39	1.5	72.6	37.8	17.2	80	1.6	46.2	23.6	10.2	/	−4.574	0.000
	C_0_‐C_2_/C_0_‐BV	39	−19.3	54.6	18.2	16.4	80	8.4	51.5	30.9	9.3	/	−4.606	0.000
	C_0_‐C_7_	39	13.5	97.8	50.7	18.5	80	25.4	80.8	49.9	11.7	/	−0.028	0.977
	C_1_‐C_7_	29	0.5	79.6	43.4	18.7	80	14.5	65.0	44.7	10.6	/	−0.096	0.924
	C_2_‐C_7_/BV‐C_7_	39	0.4	64.0	32.2	16.1	80	0.6	62.0	19.4	10.6	/	−4.297	0.000
Extension	Skull‐C_2_/Skull‐BV	37	−35.4	58.7	11.4	18.5	80	4.9	53.4	29.6	10.2	/	−5.929	0.000
	Skull‐C_7_	37	−18.8	74.8	55.3	19.0	80	24.9	87.9	66.0	11.3	/	−3.579	0.000
	C_2_‐C_7_/BV‐C_7_ wall	39	28.4	86.3	56.7	13.5	80	13.9	73.9	42.4	12.0	5.862	/	0.000
	C_0_‐C_2_/C_0_‐BV	39	−19.5	65.9	23.5	18.3	80	14.5	64.1	40.8	9.7	/	−5.359	0.000
	C_0_‐C_7_	40	0.0	109.5	73.7	20.5	80	4.7	101.7	75.4	12.6	/	−0.323	0.747
	C_1_‐C_7_	30	41.3	93.3	69.0	14.3	80	7.5	92.7	66.1	13.9	/	−0.960	0.337
	C_2_‐C_7_/BV‐C_7_	39	24.1	79.4	52.8	13.2	80	9.6	64.7	37.6	11.4	6.499	/	0.000
Flexion	Skull‐C_2_/Skull‐BV	34	−30.7	22.8	−0.7	12.3	80	0.6	29.1	9.9	5.4	/	−4.667	0.000
	Skull‐C_7_	34	−26.2	24.1	−1.3	11.9	80	−21.0	66.6	11.6	12.8	/	−4.847	0.000
	C_2_‐C_7_/BV‐C_7_ wall	40	−20.4	40.3	3.7	13.3	80	1.0	40.8	15.4	9.3	/	−4.599	0.000
	C_0_‐C_2_/C_0_‐BV	39	−17.8	58.3	14.8	17.2	80	4.9	46.9	23.2	8.2	/	−3.086	0.002
	C_0_‐C_7_	39	−11.9	57.2	16.5	13.7	80	−73.3	24.0	−2.9	16.8	/	−5.894	0.000
	C_1_‐C_7_	29	−22.5	35.5	7.6	12.7	80	−25.4	65.2	9.9	11.3	/	−0.542	0.588
	C_2_‐C_7_/BV‐C_7_	40	−23.3	36.4	2.0	15.3	80	−42.9	38.6	−7.5	22.0	/	−2.544	0.011

* The *p* values represented the statistical analyses of angles between the BI patients and control population. The C_2_‐C_7_/BV‐C_7_ wall angle and C_2_‐C_7_/BV‐C_7_ angle in extension were analyzed by Student's *t* test, while the rest of the indicators were analyzed by Mann–Whitney *U* test. *p* < 0.05 indicated a statistical difference. BV indicated block vertebra.

In the extension position, the BI patient presented smaller Skull‐C_2_/Skull‐BV angle (11.4° ± 18.5° *vs* 29.6° ± 10.2°, *P* = 0.000), Skull‐C_7_ angle (55.3° ± 19.0° *vs* 66.0° ± 11.3°, *P* = 0.000), and C_0_‐C_2_/C_0_‐BV angle (23.5° ± 18.3° *vs* 40.8° ± 9.7°, *p* = 0.000), but lager C_2_‐C_7_/BV‐C_7_ wall angle (56.7° ± 13.5° *vs* 42.4° ± 12.0°, *P* = 0.000) and C_2_‐C_7_/BV‐C_7_ angle (52.8° ± 13.2° *vs* 37.6° ± 11.4°, *P* = 0.000) than the control group.

In the flexion position, the BI patients presented smaller absolute values in Skull‐C_2_/Skull‐BV angle (−0.7° ± 12.3° *vs* 9.9° ± 5.4°, *P* = 0.000), Skull‐C_7_ angle (−1.3° ± 11.9° *vs* 11.6° ± 12.8°, *P* = 0.000), C_2_‐C_7_/BV‐C_7_ wall angle (3.7° ± 13.3° *vs* 15.4° ± 9.3°, *P* = 0.000), C_0_‐C_2_/C_0_‐BV angle (14.8° ± 17.2° *vs* 23.2° ± 8.2°, *P* = 0.002), and C_2_‐C_7_/BV‐C_7_ angle (2.0° ± 15.3° *vs* −7.5° ± 22.0°, *P* = 0.011), but lager absolute values in C_0_‐C_7_ angle (16.5° ± 13.7° *vs* −2.9° ± 16.8°, *P* = 0.000) than the control group. Among the above angles, the BI patients had a kyphotic curvature in Skull‐C_2_/Skull‐BV and Skull‐C_7_ angles, while the control group had a kyphotic curvature in C_0_‐C_7_ and C_2_‐C_7_/BV‐C_7_ angle.

**Fig. 2:**
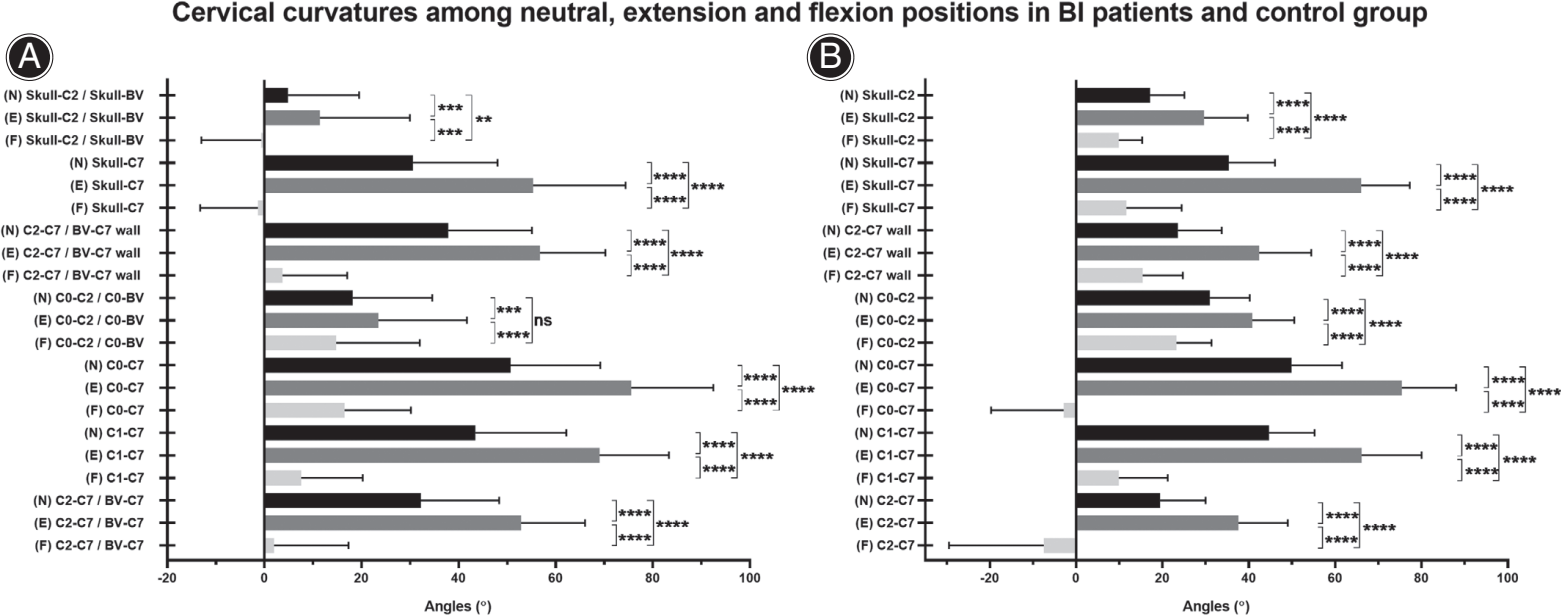
The comparison of cervical curvatures among neutral (N), extension (E), and flexion (F) positions in BI patients (A) and control group (B). The positive values indicated lordotic cervical posture, while negative values indicated kyphotic cervical posture. The order of angle's absolute values in three positions was flexion < neutral < extension. Bonferroni's multiple comparisons test was used for multiple comparison in the repeatability test. ns indicated no significant difference; ** indicated *P* < 0.01; *** indicated *P* < 0.001; **** indicated *P* < 0.0001. BV indicated block vertebra

The comparison of cervical curvature among neutral, extension, and flexion positions is shown in Figure 2. There were significant differences in all angles among different positions (all *P* < 0.0001), in both BI patients and asymptomatic people. The order of the angle's absolute values in three positions was flexion < neutral < extension. Multiple comparisons between different positions indicated that the angles in three positions were significantly different to each other in both BI patients and asymptomatic people (annotated in the figures, all *P* < 0.01 at least), except in the C_0_‐C_2_/C_0_‐BV angles between neutral and flexion positions in BI patients.

### Total, Extension, and Flexion ROMs

**Fig. 3:**
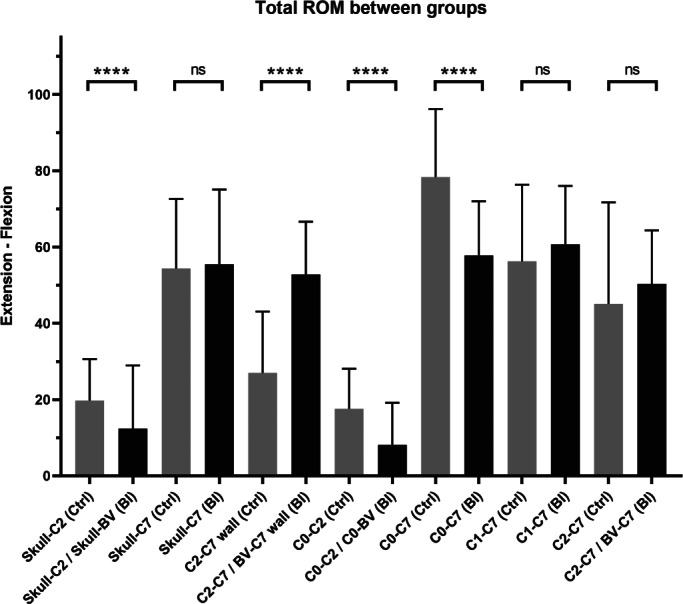
Comparison of total range of motion (ROM) between BI patients and control population. The total ROM was defined as the angle in extension position minus the angle in flexion position. The total ROM of C_2_‐ C_7_/BV‐C_7_ wall angle and total ROM of C_0_‐C_2_/C_0_‐BV angle were analyzed by Student's *t* test, while the rest of indicators were analyzed by Mann–Whitney *U* test. The BI patients presented a smaller total ROMs in Skull‐C_2_/Skull‐BV, C_0_‐C_2_/C_0_‐BV, and C_0_‐C_7_ angles but a larger total ROM in C_2_‐C_7_/BV‐C_7_ wall angle. ns indicates no significant difference; **** indicates *P* < 0.0001. BV indicated block vertebra

The results of total ROM comparison between BI patients and asymptomatic people (control group) is summarized in Figure 3 and Table 3. Generally, the total ROM of Skull‐C_2_/Skull‐BV angle and total ROM of C_0_‐C_2_/C_0_‐BV angle were smaller than the other indicators. Moreover, the BI patients presented smaller values in total ROM of Skull‐C_2_/Skull‐BV angle (12.3° ± 16.6° *vs* 19.7° ± 10.9°, *P* = 0.000), total ROM of C_0_‐C_2_/C_0_‐BV angle (8.1° ± 11.1° *vs* 17.6° ± 10.5°, *P* = 0.000), and total ROM of C_0_‐C_7_ angle (57.8° ± 14.2° *vs* 78.3° ± 17.9°, *P* = 0.000), but a larger value in total ROM of C_2_‐C_7_/BV‐C_7_ wall angle (52.8° ± 13.9° *vs* 27.0° ± 16.1°, *P* = 0.000) than the control group. No significant statistical difference was observed in the total ROM of Skull‐C_7_ angle, total ROM of C_1_‐C_7_ angle, and total ROM of C_2_‐C_7_/BV‐C_7_ angle between BI and control groups.

**TABLE 3:** Total ROM between BI and control groups

Angles	BI group					Control group					*t*	*z*	*P*‐value*
	*N*	Min	Max	Mean	SD	*N*	Min	Max	Mean	SD			
Skull‐C_2_/Skull‐BV	32	−4.7	62.5	12.3	16.6	80	−19.9	42.4	19.7	10.9	/	−4.122	0.000
Skull‐C_7_	32	7.4	87.6	55.5	19.6	80	−41.7	88.4	54.4	18.2	/	−0.586	0.558
C_2_‐C_7_/BV‐C_7_ wall	39	20.7	79.4	52.8	13.9	80	−15.4	66.6	27.0	16.1	8.594	/	0.000
C_0_‐C_2_/C_0_‐BV	38	−15.6	41.7	8.1	11.1	80	−5.9	46.4	17.6	10.5	−4.506	/	0.000
C_0_‐C_7_	38	22.1	85.7	57.8	14.2	80	37.1	126.8	78.3	17.9	/	−5.713	0.000
C_1_‐C_7_	29	28.2	104.9	60.7	15.3	80	−57.7	96.2	56.3	20.1	/	−0.833	0.405
C_2_‐C_7_/BV‐C_7_	39	22.6	77.2	50.3	14.0	80	−20.1	92.8	45.1	26.6	/	−0.413	0.679

* The *p* values represented the statistical analyses of angles between the BI patients and control population. *p* < 0.05 indicated a statistical difference. BV indicated block vertebra.

**Fig. 4:**
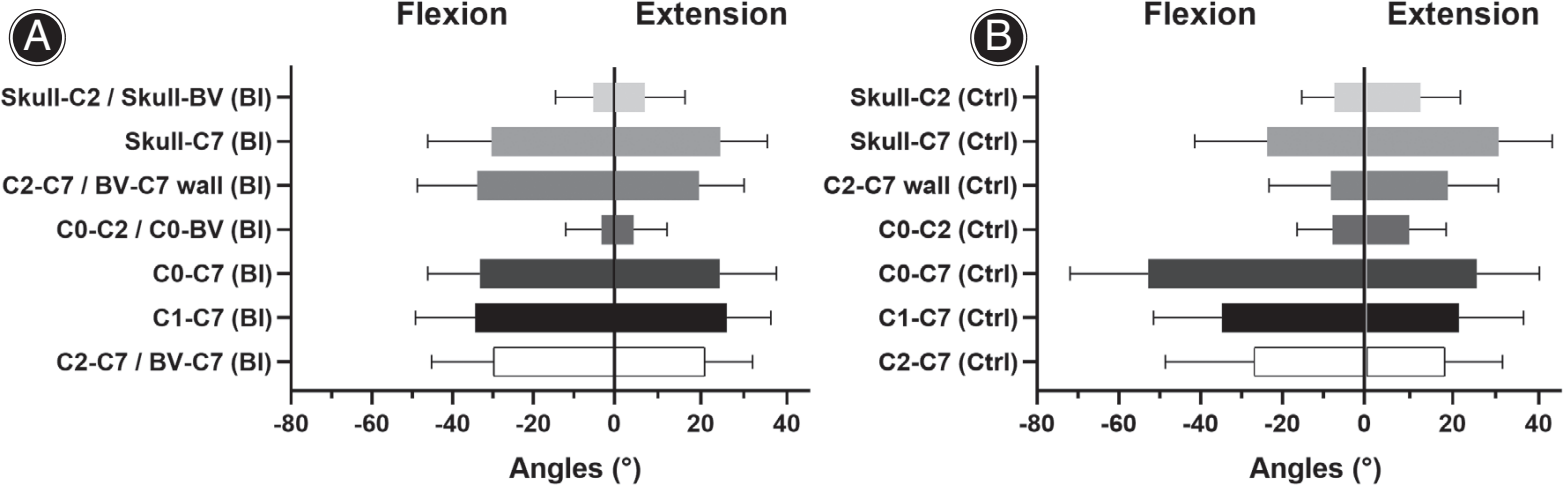
The extension and flexion ROMs of BI patients (A) and control population (B). The extension ROMs of Skull‐C_2_/Skull‐BV, C_1_‐C_7_, and C_2_‐C_7_/BV‐C_7_ angles between BI and control groups were analyzed by Mann–Whitney *U* test, while the rest of indicators were analyzed by Student's *t* test. The BI group presented smaller extension ROMs in Skull‐C_2_/Skull‐BV, Skull‐C_7_, and C_0_‐C_2_/C_0_‐BV angles. The flexion ROMs of Skull‐C_2_/Skull‐BV, C_0_‐C_7_, and C_2_‐C_7_/BV‐C_7_ angles between BI and control groups were analyzed by Mann–Whitney *U* test, while the rest of indicators were analyzed by Student's *t* test. The control group presented larger absolute values of flexion ROMs in Skull‐C_2_/Skull‐BV, C_0_‐C_2_/C_0_‐BV, and C_0_‐C_7_ angles, but a smaller absolute value of flexion ROM in C_2_‐C_7_/BV‐C_7_ wall angle. BV indicated block vertebra

The result of extension ROM between BI and control groups is presented in Figure 4 and Table 4. The extension ROM of Skull‐C_2_/Skull‐BV angle (6.9° ± 9.4° *vs* 12.5° ± 9.3°, *P* = 0.000), extension ROM of Skull‐C_7_ angle (24.5° ± 10.9° *vs* 30.7° ± 12.5°, *P* = 0.012), and extension ROM of C_0_‐C_2_/C_0_‐BV angle (4.4° ± 7.8° *vs* 9.9° ± 8.6°, *P* = 0.001) were smaller in the BI group than those in the control group. No significant difference was found in the extension ROM of C_2_‐C_7_/BV‐C_7_ wall angle, extension ROM of C_0_‐C_7_ angle, extension ROM of C_1_‐C_7_ angle, and extension ROM of C_2_‐C_7_/BV‐C_7_ angle.

**TABLE 4:** Extension ROM between BI and control groups

Angles	BI group					Control group					*t*	*z*	*P*‐value*
	*N*	Min	Max	Mean	SD	*N*	Min	Max	Mean	SD			
Skull‐C_2_/Skull‐BV	36	−4.1	46.6	6.9	9.4	80	−14.7	36.2	12.5	9.3	/	−3.870	0.000
Skull‐C_7_	36	3.6	43.6	24.5	10.9	80	−9.1	53.4	30.7	12.5	−2.555	/	0.012
C_2_‐C_7_/BV‐C_7_ wall	38	−1.8	45.4	19.6	10.5	80	−9.4	53.3	18.8	11.7	0.326	/	0.745
C_0_‐C_2_/C_0_‐BV	38	−10.0	28.7	4.4	7.8	80	−11.9	27.7	9.9	8.6	−3.361	/	0.001
C_0_‐C_7_	38	1.2	60.6	24.4	13.1	80	−46.5	53.4	25.6	14.6	−0.430	/	0.668
C_1_‐C_7_	29	1.4	51.3	26.0	10.2	80	−37.8	67.5	21.5	15.0	/	−1.670	0.095
C_2_‐C_7_/BV‐C_7_	38	5.5	50.3	20.9	11.1	80	−37.7	51.5	18.2	13.4	/	−0.780	0.435

* The *p* values represented the statistical analyses of angles between the BI patients and control population. *p* < 0.05 indicated a statistical difference. BV indicated block vertebra.

The result of flexion ROM between BI and control groups is presented in Figure 4 and Table 5. All the flexion ROMs presented negative value, indicating a forward motion of the skull and head. The BI patients showed smaller absolute values in the flexion ROM of Skull‐C_2_/Skull‐BV angle (−5.2° ± 9.4° *vs* −7.3° ± 8.0°, *P* = 0.019), flexion ROM of C_0_‐C_2_/C_0_‐BV angle (−3.2° ± 8.8° *vs* −7.7° ± 8.7°, *P* = 0.009), and flexion ROM of C_0_‐C_7_ angle (−33.2° ± 13.0° *vs* −52.8° ± 19.2°, *P* = 0.000), but a larger absolute value in flexion ROM of C_2_‐C_7_/BV‐C_7_ wall angle (−33.9° ± 14.8° *vs* −8.2° ± 15.1°, *P* = 0.000). No significant difference was found in the flexion ROM of Skull‐C_7_ angle, flexion ROM of C_1_‐C_7_ angle, and flexion ROM of C_2_‐C_7_/BV‐C_7_ angle.

**TABLE 5:** Flexion ROM between BI and control groups

Angles	BI group					Control group					*t*	*z*	*P*‐value*
	*N*	Min	Max	Mean	SD	*N*	Min	Max	Mean	SD			
Skull‐C_2_/Skull‐BV	33	−36.3	6.0	−5.2	9.4	80	−32.3	13.2	−7.3	8.0	/	−2.343	0.019
Skull‐C_7_	33	−63.6	5.4	−30.4	15.8	80	−60.0	39.8	−23.7	17.7	−1.870	/	0.064
C_2_‐C_7_/BV‐C_7_ wall	39	−71.1	−9.7	−33.9	14.8	80	−40.8	20.0	−8.2	15.1	−8.777	/	0.000
C_0_‐C_2_/C_0_‐BV	38	−28.9	15.8	−3.2	8.8	80	−24.8	12.7	−7.7	8.7	2.646	/	0.009
C_0_‐C_7_	38	−62.8	−10.9	−33.2	13.0	80	−124.5	−20.0	−52.8	19.2	/	−5.480	0.000
C_1_‐C_7_	28	−65.3	−6.8	−34.5	14.8	80	−81.6	29.1	−34.8	16.7	0.095	/	0.925
C_2_‐C_7_/BV‐C_7_	39	−68.2	7.7	−29.9	15.3	80	−59.0	29.9	−26.9	21.7	/	−0.011	0.991

* The *p* values represented the statistical analyses of angles between the BI patients and control population. *p* < 0.05 indicated a statistical difference. BV indicated block vertebra.

### Correlation Between Cobb Angles

The correlation between the Cobb angles in neutral, extension, and flexion positions were investigated in BI patients (Table S1) and control group (Table S2), respectively.

In the BI patients, the upper cervical curvature (C_0_‐C_2_/C_0_‐BV angle) was negatively correlated with the lower cervical curvature (C_2_‐C_7_/BV‐C_7_ angle) in neutral (*r* = −0.371, *P* = 0.020), extension (*r* = −0.429, *P* = 0.006), and flexion (*r* = −0.648, *P* = 0.000) positions. There was a tendency for negative correlations between the C_0_‐C_2_/C_0_‐BV and C_2_‐C_7_/BV‐C_7_ wall angles in three positions, but it was significant only in flexion position (*r* = −0.430, *P* = 0.006). The detailed correlations of the rest of the angles in the three positions is found in Table S1.

In the control group, the upper (C_0_‐C_2_ angle) and lower (C_2_‐C_7_ angle) cervical curvature tended to negatively correlate to each other in three positions, but only significantly correlated in the extension position (*r* = −0.317, *P* = 0.004). There was a tendency for negative correlations between the C_0_‐C_2_/C_0_‐BV and C_2_‐C_7_/BV‐C_7_ wall angles in the three positions, but none were significant. The detailed correlations of the rest of the angles in the three positions is found in Table S2.

## Discussion

The present study was the first study to investigate and compare the cervical curvature and the sagittal alignment change in neutral, extension, and flexion positions in BI patients and asymptomatic individuals. In the neutral position, the upper cervical curvature was smaller in BI patients (18.2°) than the asymptomatic individual (30.9°), while the lower cervical curvature was larger in BI patients (32.2°) than the control group (19.4°). The same relationship could also be observed in extension and flexion positions. The upper and lower cervical curvature presented negative correlations in neutral, extension, and flexion positions in BI patients. The total ROM of Skull‐C_2_/Skull‐BV angle, total ROM of C_0_‐C_2_/C_0_‐BV angle, and total ROM of C_0_‐C_7_ angle were smaller, while the total ROM of C_2_‐C_7_/BV‐C_7_ wall angle was larger in BI patients than these in the control group. Interestingly, the BI patients presented smaller values in extension ROM of Skull‐C_2_/Skull‐BV, extension ROM of Skull‐C_7_, and extension ROM of C_0_‐C_2_/C_0_‐BV angles, as well as smaller absolute values in flexion ROM of Skull‐C_2_/Skull‐BV, flexion ROM of C_0_‐C_2_/C_0_‐BV, and flexion ROM of C_0_‐C_7_ angles than the control group, indicating a stiffer cervical curvature.

### Cervical Curvature in BI Patients

The normal cervical curvature in asymptomatic population has been investigated in many studies, but the definitions and results varied. Inoue *et al*. defined the O‐C_2_ angle (between the McGregor line and the inferior aspect of C_2_) and the C_2_‐C_7_ angle (between the inferior aspect of the C_2_ and C_7_) as upper and lower cervical sagittal alignment, respectively. They enrolled 600 asymptomatic individuals and found the O‐C_2_ angle (14.0° lordotic) and C_2_‐C_7_ angle (14.3° lordotic) were similar in neutral position, but the mean ROM of the O‐C_2_ angle (23.1°) was smaller than ROM of C_2_‐C_7_ angle (56.0°), and the latter decreased with aging^18^. Yukawa *et al*. included 1230 asymptomatic participants, and reported a similar C_2_‐C_7_ lordosis (13.9°) in neutral position which increased with aging, and a similar C_2_‐C_7_ ROM (55.3°) which decreased with aging^19^. Liu *et al*. reported relatively larger C_2_‐C_7_ cervical curvature (21.40°) and C_2_‐C_7_ ROM (63.59°) with 300 asymptomatic volunteers^20^. In our study, the definition of Skull‐C_2_ angle (between the Chamberlain line and the inferior aspect of C_2_) was similar to the O‐C_2_ angle, and we found a similar angle value (17.1° lordotic) and corresponding total ROM (19.7°) in the control group. However, the BI patients had a notably smaller Skull‐C_2_/Skull‐BV angle (4.9° lordotic) and corresponding total ROM (12.3°), indicating that their skull‐cervical relationship is stiffer than the asymptomatic individuals. Generally compared with the reported study, we observed a relatively larger C_2_‐C_7_ angle (19.4° lordotic) and smaller C_2_‐C_7_ total ROM (45.1°) in the control group. On the contrary, the BI patients presented significantly larger C_2_‐C_7_/BV‐C_7_ angle (32.2° lordotic) but a smaller C_2_‐C_7_/BV‐C_7_ total ROM (50.3°) than the asymptomatic population from the literature.

Lee *et al*. divided the cervical lordosis into upper (C_0_‐C_2_ angle, defined as angle between McRae line and the C_2_ lower end plate) and lower ones (C_2_‐C_7_ wall angle, defined as angle between the posterior walls of the C_2_ and the C_7_ vertebral bodies) in 77 asymptomatic adults, and found that the C_0_‐C_2_ angle was 22.4° lordotic while the C_2_‐C_7_ wall angle was 9.9° lordotic^21^. Iyer *et al*. had the same definitions and reported the angles with 21.6° lordotic for C_0_‐C_2_ angle and 13.7° lordotic for C_2_‐C_7_ wall angle^22^. However, the result of the present study showed that in neutral position, the control group had a larger C_0_‐C_2_ angle (30.9°) and C_2_‐C_7_ wall angle (23.6°), while the BI patients presented a lower C_0_‐C_2_/C_0_‐BV angle (18.2°) but a greater C_2_‐C_7_/BV‐C_7_ wall angle (37.8°). The aforementioned studies were conducted on Koreans and Americans while we investigated a Chinese population. Hence, the inconsistency of results might be due to racial difference.

### Negative Correlations Between Angles

It was revealed by Inoue *et al*. that the O‐C_2_ angle was negatively correlated with the C_2_‐C_7_ angle in asymptomatic population^18^. The same negative correlation result between upper and lower cervical alignments was reported in another study of an Indian population^23^. In the present study, we observed that the trend of negative correlation existed not only between the C_0_‐C_2_/C_0_‐BV and C_2_‐C_7_/BV‐C_7_ angles, but also between the C_0_‐C_2_/C_0_‐BV and C_2_‐C_7_/BV‐C_7_ wall angles, in both the control group and BI patients. Interestingly, such relation was more obvious in the BI population, because its upper and lower cervical curvatures negatively and significantly correlated with each other in all positions, while the significant result was observed only in the extension position in the control group. In general, in order to maintain a horizontal eyesight, if the upper curvature grew greater, the lower curvature would become smaller, and vice versa. The abnormal anatomic structure, especially the CVJ region in the BI patients, caused the chaos of cervical alignment. Prolapse of the odontoid process into the foramen magnum led to relative downward angle of skull to the cervical spine. In addition, it was reported that the sagittal inclination was increased in BI patients compared to the non‐deformity subjects^24^, which also indicated the downward angle of skull. Therefore, it was reasonable to find that the BI patients had a smaller upper cervical curvature but a larger lower curvature comparing with the control group.

The cervical curvature was regulated by muscles, ligaments, and skeletons. Normally, when the curvature changed, the muscles and ligaments were used for compensation before the skeletons. If the changes were less apparent, compensation of soft tissues without skeletons would be enough. For asymptomatic population, the change of Cobb angles were more apparent in extension position, necessitating the skeleton compensation so that negative correlation was more obvious in extension position rather than in neutral and flexion positions. For the BI patients, the abnormal bony structures caused an insufficient compensatory ability. Once the upper cervical curvature changed greatly, the lower cervical curvature would quickly and apparently present a negative correlation change. Thus, the statistical differences were observed in all three positions.

### Total Cervical Curvature

Apart from the aforementioned angles, the total cervical curvature was a concerning indicator. Jouibari *et al*. reported that the C_1_‐C_7_ angle was 45.7° in the non‐symptom group^25^, which was comparable to our finding in the control group (44.7°) and the BI patients (43.4°) in the neutral position. We also measured the Skull‐C_7_ angle and found that the results were comparable in neutral position between the two groups, but there was not relative reporting of this in other studies as far as we knew. We believed that the structural disorder of BI patients mainly involved the relative position of the C_2_ odontoid process. Even though some of the patients were found to have a fusion between C_0_ and C_1_, the height of anterior and posterior arches were too short to influence the results of C_0_‐C_7_ and C_1_‐C_7_ angles significantly. In addition, the hard palate was not affected as much as the C_2_ odontoid process by the disease, so that the Skull‐C_7_ angles did not dramatically differ between the BI patients and asymptomatic individuals. However, the above discussion was only applicable in neutral and extension positions. Because the flexion position could force the odontoid process to prolapse backward and upward relatively, it would make the abnormal structures more complicated so that it was difficult to find the relationship and connections between groups.

### Effect of Symptoms and Sub‐types

The BI patients commonly co‐occurred with symptoms such as weakness in limbs, neck pain, and gait disturbance^26^. We assume that the BI patients with a more severe pain would associate with larger cervical curvature. Besides, the pain would prevent them from hyperactivity, which will affect the measurement results of curvature. However, as far as we know, the relationship between curvature and severity of pain in BI patients has not been reported. This is a retrospective study based on the outpatient department and the results were mainly analyzed from radiological images. We did not include the symptoms into the analysis. We would continue to investigate this problem in our future research.

According to Goel's classification, the BI patients could be divided into Group I and II according to the absence or presence of Chiari malformation^4^, or be divided into Group A and B according to the relative positions between the odontoid process and Chamberlain, McRae, and Wackenheim lines^5^. The cervical curvature was defined by the angles between various lines in this study. The atlantoaxial dislocation or instability would lead to a change of the anatomic structures, and affect the lines such as the Chamberlain line, C_0_ line, C_1_ line, and C_2_ line, so the angles could be influenced. To be specific (assuming no bony fusion presented), in Group A with the prolapse of C_2_ and its distancing from anterior arch, the clivus and anterior arch relatively moved downward, so that the most affected parameters would be C_0_ and C_1_ lines, and their related angles would be smaller and even become kyphotic. In Group B, the odontoid process, anterior arch, and the clivus migrated superiorly together and the clivus is more flat. So the relative angles of C_0_ and C_1_ lines would become larger.

### Clinical Relevance of Cervical Alignment

The cervical alignment is an important factor in the reconstruction of the CVJ. Many studies have revealed the correlation between cervical alignment parameters and the health‐related quality of life (HRQOL) outcomes such as the neck disability index (NDI)^22^, ^27^, ^28^, ^29^. It was suggested that the positive sagittal malalignment in cervical spine after surgical treatment would increase the risks of severe disability^30^. Therefore, for the BI patients, apart from selecting appropriate surgical approach, fixation method, and postoperative care, the restoring of cervical alignment should be emphasized. A successful surgery should regain not only the structural stabilization but also proper cervical curvature. The results of cervical alignment in BI patients in the present study could be provided as reference for the surgeons. However, it has been pointed out that there is not consensus on optimal amount of postoperative cervical lordosis to be achieved, and the general principle is to reconstruct the cervical curvature as close to neutral position as possible^6^. In addition, it should be noted that kyphotic cervical posture could also be observed in normal healthy adults^19^, ^31^. It was found that the negative correlation between upper and lower curvature was still applicable in the BI patients in this study, indicating that although the angles might change in BI patients, the relationship of upper and lower curvatures remained the same with that of asymptomatic population.

In our previous research, the characteristics of the cranial‐cervical spinal canal^16^, the thickness of the occipital bone^17^, and the anatomic feature of clivus with atlas assimilation^32^ were investigated in patients with congenital malformation in CVJ region. The present study described the cervical curvature and the ROMs of relative parameters in BI patients, supplementing the lack of the morphological feature of cervical alignment in this population. Together with our previous work, it could serve as reference to the future application of the clivus plate fixation system in the BI population.

## Limitations

There were some limitations in this study. Firstly, the sample size of the BI patients was small since they were not as common as the asymptomatic population, and not all the cases met the inclusion criteria. However, it is necessary to summarize the characteristics of such rare and precious cases as a reference for future clinic practice. Secondly, the present study did not investigate the effect of aging, gender, and subtypes. It was reported that some cervical alignment parameters were related to these factors in asymptomatic subjects^18^, ^19^, ^20^. According to Geol's classification^4^, ^5^, the sample size of Group B was too small so far, hence we did not further divided these 40 cases into subtypes to compare their differences in cervical curvature. With more BI cases being accessible in the future, we can include these factors in our next study and discuss their effect on cervical alignment parameters in the BI population. Thirdly, we did not include other spine‐ and pelvis‐related parameters in this study, such as T_1_ slope and pelvic incidence, which are getting more and more attention in sagittal balance. We could analyze more information about the spinal alignment of BI patients if we could access the radiological images of the whole spine and pelvis in future studies.

## Conclusion

The present study firstly described the cervical curvature and ROM of cervical alignment parameters in neutral, extension, and flexion positions in patients with BI, finding that (i) overall, the cervical spine was stiffer in BI patients than the asymptomatic individuals, especially in the upper cervical curvature, and (ii) the negative correlation between upper and lower cervical curvatures was more obvious in BI patients.

## Supporting information


**Appendix S1**: **Table S1:** Correlation between angles in three postures in BI patients
**Table S2:** Correlation between angles in three postures in control group
