# Supplementary material for: Cervical Alignment of Patients with Basilar Invagination: A Radiological Study
Source: Orthop Surg. 2022 Feb 13;14(3):566–76. doi: 10.1111/os.13212 (PMC8926990; doi:10.1111/os.13212)
Supplement: Supplementary file 1 — Appendix S1: Table S1: Correlation between angles in three postures in BI patients Table S2: Correlation between angles in three postures in control group [file OS-14-566-s001.doc]

| **Supplemental table 1 Correlation between Angles in three postures in BI patients** | | | | | | | | |
| --- | --- | --- | --- | --- | --- | --- | --- | --- |
|  |  | Neutral | | | | | | |
|  |  | Skull-C2 / Skull-BV | Skull-C7 | C2-C7 / BV-C7 wall | C0-C2 / C0-BV | C0-C7 | C1-C7 | C2-C7 / BV-C7 |
| Neutral | Skull-C2 / Skull-BV | 1.000 | -0.087 | -0.166 | 0.458 | 0.240 | 0.186 | -0.196 |
|  |  |  | 0.599 | 0.311 | 0.003 | 0.141 | 0.333 | 0.232 |
|  | Skull-C7 |  | 1.000 | 0.413 | 0.236 | 0.531 | 0.538 | 0.346 |
|  |  |  |  | 0.009 | 0.148 | 0.001 | 0.003 | 0.031 |
|  | C2-C7 / BV-C7 wall |  |  | 1.000 | -0.076 | 0.624 | 0.659 | 0.772 |
|  |  |  |  |  | 0.645 | 0.000 | 0.000 | 0.000 |
|  | C0-C2 / C0-BV |  |  |  | 1.000 | 0.568 | 0.522 | -0.371 |
|  |  |  |  |  |  | 0.000 | 0.004 | 0.020 |
|  | C0-C7 |  |  |  |  | 1.000 | 0.875 | 0.545 |
|  |  |  |  |  |  |  | 0.000 | 0.000 |
|  | C1-C7 |  |  |  |  |  | 1.000 | 0.535 |
|  |  |  |  |  |  |  |  | 0.003 |
|  | C2-C7 / BV-C7 |  |  |  |  |  |  | 1.000 |
|  |  | Extension | | | | | | |
|  |  | Skull-C2 / Skull-BV | Skull-C7 | C2-C7 / BV-C7 wall | C0-C2 / C0-BV | C0-C7 | C1-C7 | C2-C7 / BV-C7 |
| Extension | Skull-C2 / Skull-BV | 1.000 | -0.239 | -0.172 | 0.390 | 0.121 | 0.078 | -0.324 |
|  |  |  | 0.155 | 0.308 | 0.017 | 0.476 | 0.688 | 0.050 |
|  | Skull-C7 |  | 1.000 | 0.078 | 0.411 | 0.421 | 0.285 | 0.044 |
|  |  |  |  | 0.647 | 0.011 | 0.010 | 0.134 | 0.795 |
|  | C2-C7 / BV-C7 wall |  |  | 1.000 | -0.145 | 0.414 | 0.500 | 0.683 |
|  |  |  |  |  | 0.378 | 0.009 | 0.005 | 0.000 |
|  | C0-C2 / C0-BV |  |  |  | 1.000 | 0.699 | 0.436 | -0.429 |
|  |  |  |  |  |  | 0.000 | 0.016 | 0.006 |
|  | C0-C7 |  |  |  |  | 1.000 | 0.724 | 0.293 |
|  |  |  |  |  |  |  | 0.000 | 0.070 |
|  | C1-C7 |  |  |  |  |  | 1.000 | 0.298 |
|  |  |  |  |  |  |  |  | 0.110 |
|  | C2-C7 / BV-C7 |  |  |  |  |  |  | 1.000 |
|  |  | Flexion | | | | | | |
|  |  | Skull-C2 / Skull-BV | Skull-C7 | C2-C7 / BV-C7 wall | C0-C2 / C0-BV | C0-C7 | C1-C7 | C2-C7 / BV-C7 |
| Flexion | Skull-C2 / Skull-BV | 1.000 | 0.089 | -0.405 | 0.429 | 0.143 | 0.306 | -0.279 |
|  |  |  | 0.615 | 0.017 | 0.011 | 0.421 | 0.137 | 0.110 |
|  | Skull-C7 |  | 1.000 | 0.388 | -0.170 | 0.299 | 0.113 | 0.546 |
|  |  |  |  | 0.023 | 0.336 | 0.086 | 0.590 | 0.001 |
|  | C2-C7 / BV-C7 wall |  |  | 1.000 | -0.430 | 0.403 | 0.407 | 0.730 |
|  |  |  |  |  | 0.006 | 0.011 | 0.028 | 0.000 |
|  | C0-C2 / C0-BV |  |  |  | 1.000 | 0.504 | 0.337 | -0.648 |
|  |  |  |  |  |  | 0.001 | 0.074 | 0.000 |
|  | C0-C7 |  |  |  |  | 1.000 | 0.711 | 0.219 |
|  |  |  |  |  |  |  | 0.000 | 0.181 |
|  | C1-C7 |  |  |  |  |  | 1.000 | 0.234 |
|  |  |  |  |  |  |  |  | 0.222 |
|  | C2-C7 / BV-C7 |  |  |  |  |  |  | 1.000 |

Note: The values of each cell indicated the Pearson correlation coefficient (upper) and p value (lower), respectively. p < 0.05 indicated a statistical difference. BV indicated block vertebra.

| **Supplemental table 2 Correlation between Angles in three postures in control group** | | | | | | | | |
| --- | --- | --- | --- | --- | --- | --- | --- | --- |
|  |  | Neutral | | | | | | |
|  |  | Skull-C2 | Skull-C7 | C2-C7 wall | C0-C2 | C0-C7 | C1-C7 | C2-C7 |
| Neutral | Skull-C2 | 1.000 | 0.469 | -0.019 | 0.618 | 0.320 | 0.170 | -0.238 |
|  |  |  | 0.000 | 0.865 | 0.000 | 0.004 | 0.133 | 0.033 |
|  | Skull-C7 |  | 1.000 | 0.701 | 0.254 | 0.779 | 0.706 | 0.591 |
|  |  |  |  | 0.000 | 0.023 | 0.000 | 0.000 | 0.000 |
|  | C2-C7 wall |  |  | 1.000 | -0.123 | 0.539 | 0.633 | 0.632 |
|  |  |  |  |  | 0.276 | 0.000 | 0.000 | 0.000 |
|  | C0-C2 |  |  |  | 1.000 | 0.622 | 0.039 | -0.198 |
|  |  |  |  |  |  | 0.000 | 0.728 | 0.078 |
|  | C0-C7 |  |  |  |  | 1.000 | 0.566 | 0.481 |
|  |  |  |  |  |  |  | 0.000 | 0.000 |
|  | C1-C7 |  |  |  |  |  | 1.000 | 0.529 |
|  |  |  |  |  |  |  |  | 0.000 |
|  | C2-C7 |  |  |  |  |  |  | 1.000 |
|  |  | Extension | | | | | | |
|  |  | Skull-C2 | Skull-C7 | C2-C7 wall | C0-C2 | C0-C7 | C1-C7 | C2-C7 |
| Extension | Skull-C2 | 1.000 | 0.405 | -0.250 | 0.656 | 0.150 | -0.098 | -0.404 |
|  |  |  | 0.000 | 0.025 | 0.000 | 0.184 | 0.389 | 0.000 |
|  | Skull-C7 |  | 1.000 | 0.615 | 0.307 | 0.724 | 0.728 | 0.570 |
|  |  |  |  | 0.000 | 0.006 | 0.000 | 0.000 | 0.000 |
|  | C2-C7 wall |  |  | 1.000 | -0.174 | 0.475 | 0.795 | 0.844 |
|  |  |  |  |  | 0.123 | 0.000 | 0.000 | 0.000 |
|  | C0-C2 |  |  |  | 1.000 | 0.409 | 0.025 | -0.317 |
|  |  |  |  |  |  | 0.000 | 0.825 | 0.004 |
|  | C0-C7 |  |  |  |  | 1.000 | 0.741 | 0.476 |
|  |  |  |  |  |  |  | 0.000 | 0.000 |
|  | C1-C7 |  |  |  |  |  | 1.000 | 0.751 |
|  |  |  |  |  |  |  |  | 0.000 |
|  | C2-C7 |  |  |  |  |  |  | 1.000 |
|  |  | Flexion | | | | | | |
|  |  | Skull-C2 | Skull-C7 | C2-C7 wall | C0-C2 | C0-C7 | C1-C7 | C2-C7 |
| Flexion | Skull-C2 | 1.000 | 0.176 | 0.355 | 0.488 | -0.344 | 0.301 | -0.034 |
|  |  |  | 0.119 | 0.001 | 0.000 | 0.002 | 0.007 | 0.768 |
|  | Skull-C7 |  | 1.000 | 0.489 | 0.016 | -0.342 | 0.714 | -0.170 |
|  |  |  |  | 0.000 | 0.889 | 0.002 | 0.000 | 0.132 |
|  | C2-C7 wall |  |  | 1.000 | 0.206 | -0.189 | 0.316 | -0.364 |
|  |  |  |  |  | 0.067 | 0.094 | 0.004 | 0.001 |
|  | C0-C2 |  |  |  | 1.000 | -0.200 | 0.029 | -0.055 |
|  |  |  |  |  |  | 0.075 | 0.800 | 0.628 |
|  | C0-C7 |  |  |  |  | 1.000 | -0.481 | 0.035 |
|  |  |  |  |  |  |  | 0.000 | 0.759 |
|  | C1-C7 |  |  |  |  |  | 1.000 | -0.125 |
|  |  |  |  |  |  |  |  | 0.271 |
|  | C2-C7 |  |  |  |  |  |  | 1.000 |

Note: The values of each cell indicated the Pearson correlation coefficient (upper) and p value (lower), respectively. p < 0.05 indicated a statistical difference.
